# Cytostatic Factor Proteins Are Present in Male Meiotic Cells and β-Nerve Growth Factor Increases Mos Levels in Rat Late Spermatocytes

**DOI:** 10.1371/journal.pone.0007237

**Published:** 2009-10-05

**Authors:** Marie-Hélène Perrard, Emeric Chassaing, Guillaume Montillet, Odile Sabido, Philippe Durand

**Affiliations:** 1 Institut de génomique fonctionnelle de Lyon, Université de Lyon, INRA UMR 1288, CNRS UMR 5242, Université Lyon 1, Ecole Normale Supérieure de Lyon, Lyon, France; 2 Université Jean Monnet, Faculté de médecine, Centre commun de Cytométrie en flux, St-Etienne, France; Cincinnati Children's Research Foundation, United States of America

## Abstract

**Background:**

In co-cultures of pachytene spermatocytes with Sertoli cells, β-NGF regulates the second meiotic division by blocking secondary spermatocytes in metaphase (metaphase II), and thereby lowers round spermatid formation. In vertebrates, mature oocytes are arrested at metaphase II until fertilization, because of the presence of cytostatic factor (CSF) in their cytoplasm. By analogy, we hypothesized the presence of CSF in male germ cells.

**Methodology/Principal Findings:**

We show here, that Mos, Emi2, cyclin E and Cdk2, the four proteins of CSF, and their respective mRNAs, are present in male rat meiotic cells; this was assessed by using Western blotting, immunocytochemistry and reverse transcriptase PCR. We measured the relative cellular levels of Mos, Emi2, Cyclin E and Cdk2 in the meiotic cells by flow cytometry and found that the four proteins increased throughout the first meiotic prophase, reaching their highest levels in middle to late pachytene spermatocytes, then decreased following the meiotic divisions. In co-cultures of pachytene spermatocytes with Sertoli cells, β-NGF increased the number of metaphases II, while enhancing Mos and Emi2 levels in middle to late pachytene spermatocytes, pachytene spermatocytes in division and secondary spermatocytes.

**Conclusion/Significance:**

Our results suggest that CSF is not restricted to the oocyte. In addition, they reinforce the view that NGF, by enhancing Mos in late spermatocytes, is one of the intra-testicular factors which adjusts the number of round spermatids that can be supported by Sertoli cells.

## Introduction

Spermatogenesis is a complex process during which diploid spermatogonia divide mitotically to provide a population of spermatocytes that proceed through meiosis to haploid spermatids which differenciate into spermatozoa [1]. Multiplication, differentiation and survival or death of testicular germ cells are tightly regulated by both endocrine and local interactions: in addition to the regulation exerted by FSH and LH, spermatogenesis requires testicular factors originating from somatic and/or germ cells [1], [2]. This process occurs in seminiferous tubules, where germ cells are nursed by Sertoli cells [1], [2]. Each Sertoli cell is able to support a limited number of germ cells, in a species-specific manner [3]. We have previously shown that β-Nerve growth factor (β-NGF) participates in the regulation of spermatocyte differentiation by blocking secondary spermatocytes (SII) in metaphase (MPII) leading to a reduction of round spermatid (RS) formation in co-cultures of pachytene spermatocytes (PS) with Sertoli cells [4].

Masui and Market [5] postulated the presence of a specific factor in the cytoplasm of the oocyte blocked in MPII, and named this factor “cytostatic factor” (CSF). Sagata et al [6] proposed that the proto-oncogene Mos, a germ-cell-specific protein kinase uniquely induced at the beginning of oocyte maturation, was responsible for this CSF arrest in Xenopus eggs. Subsequently, the mitogen-activated protein kinase (MAPK) pathway comprising the mitogen-activated protein kinase kinase (MEK) and extracellular signal-regulated kinases (Erk) 1/2 were identified as the downstream mediators of Mos [7]. Recently, it has been shown that one early mitotic inhibitor (Emi) protein, Emi2/Fbxo43 is required for both the establishment and maintenance of CSF arrest in Xenopus and mice eggs and that the Mos-MAPK pathway is directly linked to Emi2 for both CSF establishment and maintenance [8], [9]. An independent pathway involving Cdk2-Cyclin-E was also characterized through an antisense oligonucleotide approach [10], although these results have subsequently been challenged by the observation that injection of the Cdk2 inhibitor p21^CIP^ does not interfere with CSF arrest [11]. Therefore, we hypothesized the presence of CSF in male germ cells, which could be, at least in part, responsible for the β-NGF induced blockage of SII in MPII observed in co-cultures of PS with Sertoli cells. We have shown previously, that both ERK1 and ERK2 are detected in meiotic cells, from young PS to RS, present in co-culture of PS with Sertoli cells, as in freshly isolated germ cell populations. Moreover, their activated (phosphorylated) forms increased during meiotic progression, suggesting a role of MAPKs in this process [12]. However informations on the proteins constitutive of CSF and their role, if any, are scarce in the male.

In the first part of the present study we highlighted the presence of Mos, Emi2, cyclin E and Cdk2 proteins, and their respective mRNAs in freshly isolated male rat meiotic cells and determined the relative cellular levels in these proteins throughout the meiotic phase. In the second part, we addressed the effect of β-NGF on Mos, Emi2, cyclin E and Cdk2 protein levels in cultured meiotic cells. It was found that β-NGF increased Mos and Emi2 levels in middle to late pachytene spermatocytes, pachytene spermatocytes in division and secondary spermatocytes.

## Results

### Components of CSF are present in male germ cells

#### RT-PCR

**Figure 1:**
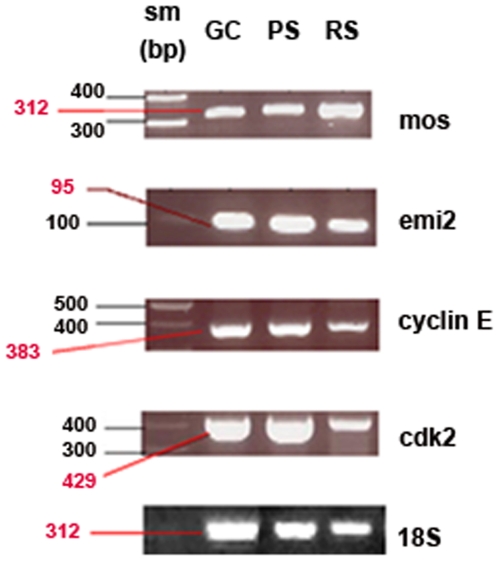
Expression of the mRNAs of mos, emi2, cyclin E and cdk2 in total germ cells (GC), PS and RS. 18S served as a loading control. Specific mRNA/18S mRNA ratios are indicated under each band. sm (size marker).

The mRNAs encoding Mos, Emi2, Cyclin E and Cdk2 were detected at the expected sizes by RT-PCR in freshly prepared total germ cells and elutriated PS and RS (Fig. 1). By measuring the mos or emi2 or cyclin E or cdk2 mRNA/18S RNA ratios it was found that the amount of mos mRNA was about two fold higher in RS than in PS, while emi2 mRNA and cyclin E mRNA amounts were roughly similar in PS and RS. The amount of cdk2 mRNA was a little higher in PS than in RS.

#### Western blotting

**Figure 2:**
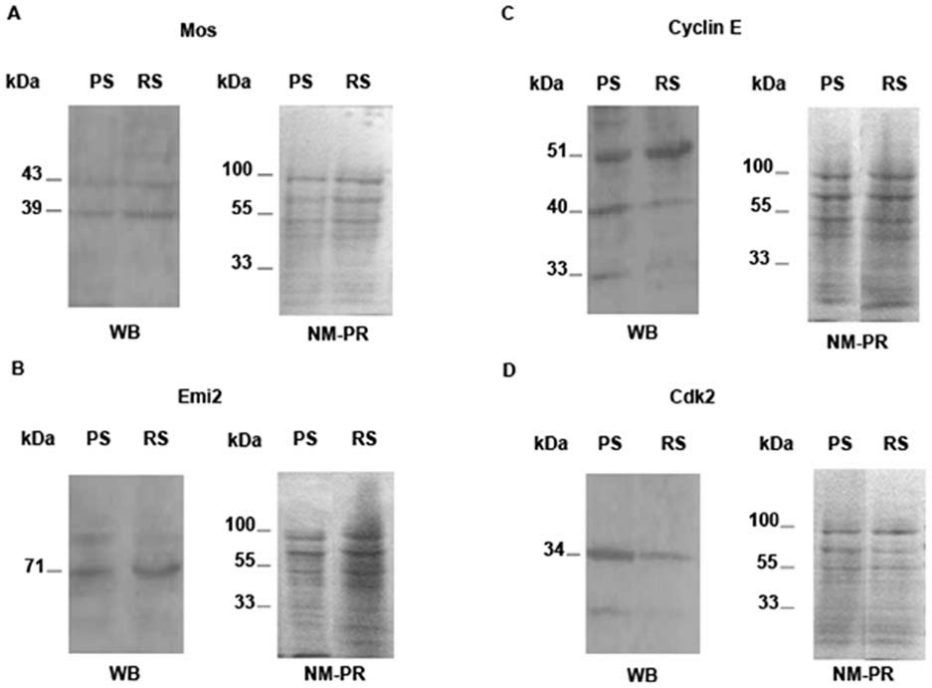
Detection of Mos (A), Emi2 (B), Cyclin E (C) and Cdk2 (D) by Western blotting in PS and RS. Fifty µg of PS and RS were resolved by SDS-PAGE (10% gel). For each protein are presented (i) the Western blot (WB) highlighting the specific bands: 39 and 43 kDa for Mos, 71 kDa for Emi2, 51 kDa for Cyclin E (40 and 33 kDa are low molecular weight derivatives) and 34 kDa for Cdk2, and (ii) the staining of the nitrocellulose membranes with Ponceau Red S (NM-PR) assessing the blotting of proteins. The molecular weights of each specific band are indicated on the left of the WB. Locations of molecular mass standards are shown on the left of NM-PR.

The specificity of the commercial anti-cyclin E, anti-Cdk2, anti-Mos and anti-Emi2 antibodies was tested by Western blotting (Fig. 2). The anti-Mos antibody detected two immunoreactive bands of protein in both the PS and RS fractions (Fig. 2A): (i) the 43 kDa band, which was previously described in the mouse and rat testes [13], [14] and (ii) the 39 kDa band which was shown in Xenopus, mouse and bovine oocytes [15]–[17]. Fig. 2B shows a western blot of Emi2 in elutriated PS and RS fractions. The 71 kDa band representing Emi2 in the mouse testis [9] was expressed in the two cell types. The 51 kDa band corresponding to Cyclin E [18] and low molecular weight derivatives [19] were observed in both PS and RS (Fig. 2C). The anti-Cdk2 antibody detected one band of protein at the expected size of about 34 kDa in PS and RS fractions (Fig. 2D). As expected staining of the corresponding nitrocellulose membrane with Ponceau Red S showed differences in blotted proteins between the two cell types but ascertained protein transfer. Since Western Blotting is a semi-quantitative method, the accurate cellular quantification by flow cytometry [12], [20], [21] of Mos, Emi2, Cyclin E and Cdk2 in meiotic cells was performed (see below).

#### Immunocytochemistry

**Figure 3:**
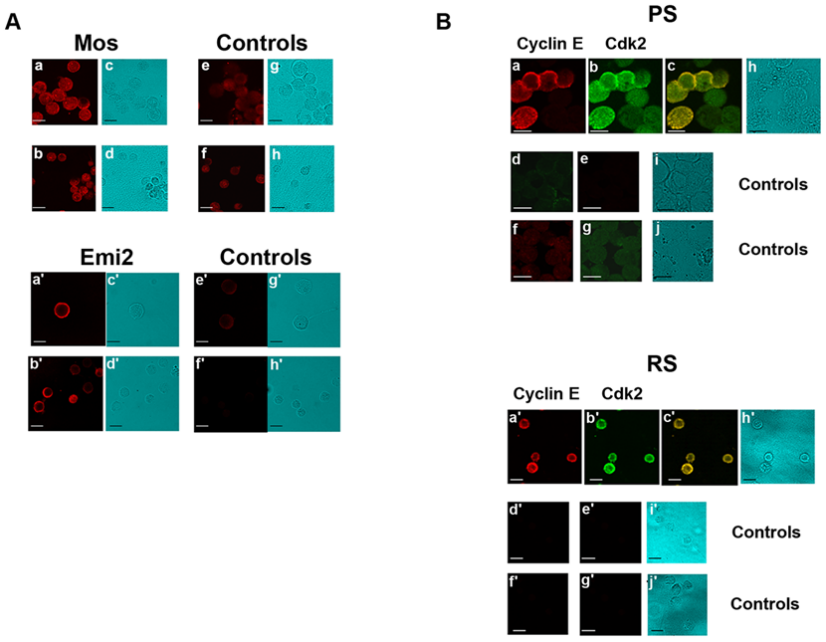
Fluorescent immunocytochemical staining of Mos, Emi2, CyclinE and Cdk2 in elutriated PS and RS. A) Immunocytochemical localization of Mos and Emi2 in PS and RS. Mos and Emi2 are present in PS (a, a′), and in RS (b, b′). e, f (reaction with non immune rabbit IgG) and e′, f′ (reaction with non immune goat IgG) are the corresponding controls. c, d, g, h, c′, d′, g′ and h′ are the transmission pictures corresponding to a, b, e, f, a′, b′, e′ and f′ respectively. B): Immunocytochemical co-localization of Cyclin E and Cdk2 in PS and RS. Cyclin E (a, a′) and Cdk2 (b, b′) are present in PS and RS (c, c′: merge) respectively. d, d′, e and e′ are controls for Cdk2 (reaction with non immune mouse IgG) which were examined for green or red fluorescence. f, f′, g and g′ are controls for Cyclin E (reaction with non immune rabbit IgG) which were examined for red or green fluorescence. h and h′ are the transmission pictures corresponding to a, b, c and a′, b′, c′ respectively. i and i′ correspond to d, e and d′, e′; j and j′ correspond to f, g and f′, g′ respectively. All bars represent 10 µm.

Fluorescent immunocytochemical staining of Mos, Emi2, CyclinE and Cdk2 showed that these proteins were detected in elutriated PS and RS (Fig. 3); the labeling appeared localized mostly in the cytoplasm.

#### Flow cytometry

**Figure 4:**
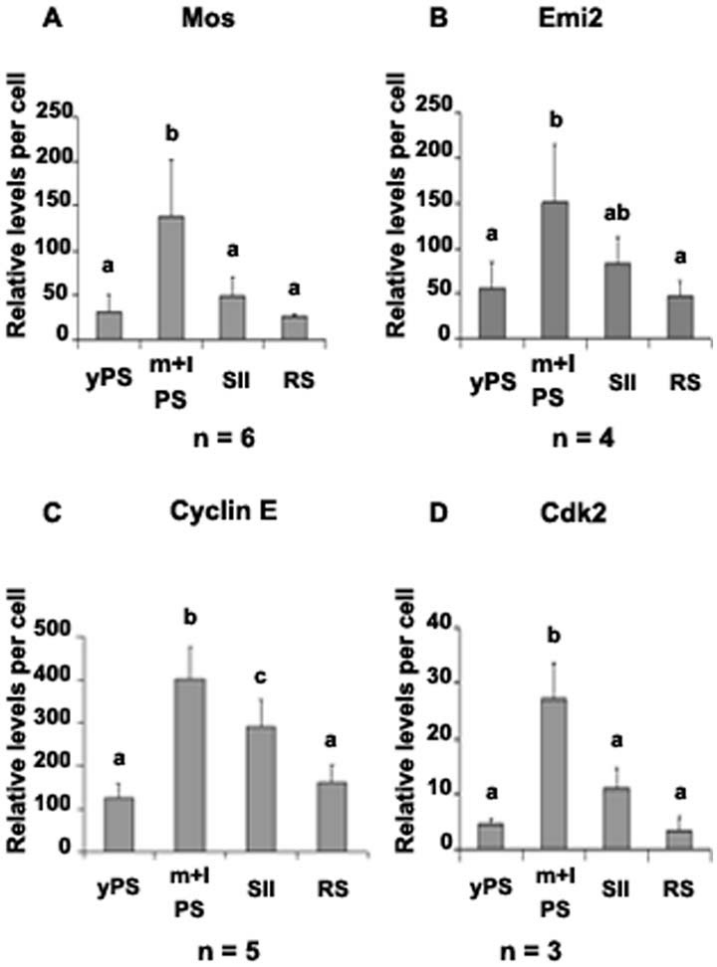
Relative levels of Mos (A) or Emi2 (B) or Cyclin E (C) or Cdk2 (D) in the different populations of rat testis meiotic cells (young PS (yPS), middle to late PS (m+lPS), SII, RS). Each point is the mean±SEM of n experiments. Different letters indicate statistically significant differences (p<0.05 or p<0.01).

Four populations of male rat meiotic cells (young PS, middle to late PS, SII and RS) were identified by cytometry [12]. The relative levels of Mos, Emi2, Cyclin E and Cdk2 were measured in these populations. For every protein, low levels were observed in young PS. These levels increased during the first meiotic prophase, reaching their highest levels in middle to late PS, then decreased following the meiotic divisions (Fig. 4).

Taken together these results indicate that the main components of CSF identified so far in the oocyte are present in rat male meiotic cells. Moreover it appears that the cell contents in these proteins culminate just before the first meiotic division.

### Regulation of Mos and Emi2 proteins by β-NGF

**Figure 5:**
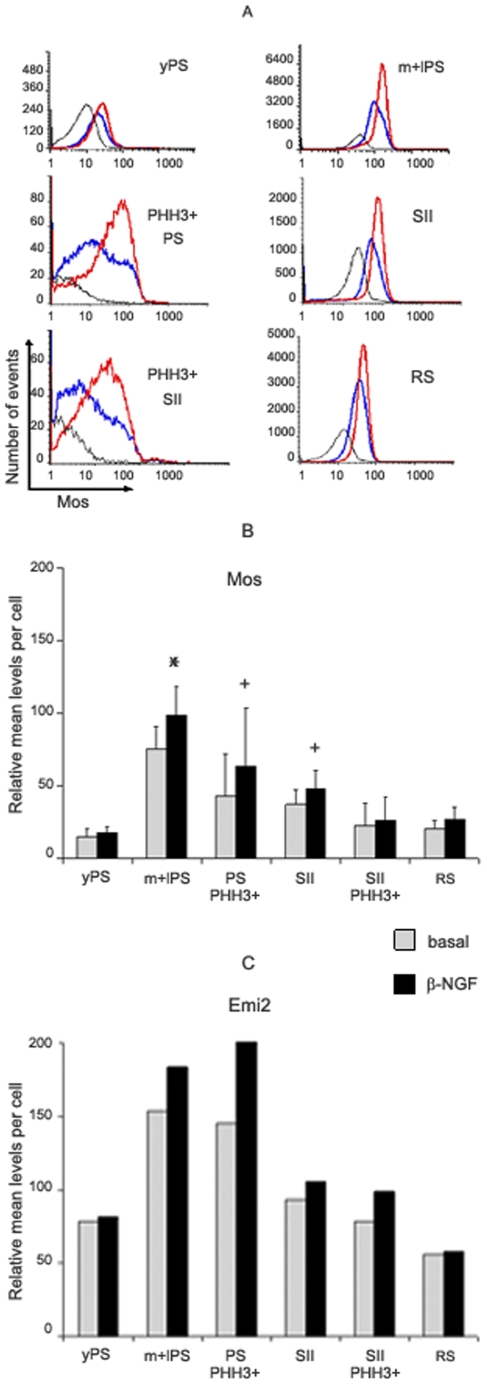
β-NGF induced changes in Mos and Emi2 levels in co-cultured meiotic cells. 5A: Relative levels of Mos in young PS (yPS), middle to late PS (m+lPS), PS in division (PHH3+ PS), SII, SII in division (PHH3+ SII) and RS populations co-cultured with Sertoli cells for three days under basal condition (no β-NGF added: blue line), or in the presence of β-NGF(10 ng/ml: red line). Co-cultured cells were incubated with non immune rabbit IgG as a control (black line). Analysis was performed on 1.42×10^6^, 1.54×10^6^ and 1.80×10^6^ cells in control, basal and β-NGF treated cells respectively. Note the logarithmic scale of the x axis. 5B: Changes in the mean levels of Mos in the different populations of cultured meiotic cells under basal conditions or in presence of β-NGF. Each point is the mean±SEM of 5 experiments.* p≤0.02 vs control + p≤0.05 vs control 5C: Changes in the mean levels of Emi2 in the different populations of cultured meiotic cells under basal conditions or in presence of β-NGF. Results are from one representative experiment. Similar results were obtained in two other experiments.

We investigated next whether β-NGF led to some changes in either Mos or Emi2 or Cyclin E or Cdk2 levels in meiotic cells. PS were co-cultured for three days with Sertoli cells either in the absence or presence of β-NGF and the cellular contents in Mos, Emi2, Cyclin E and Cdk2 were measured in young PS, middle to late PS, PS in division, SII, SII in division and RS. Under basal conditions (no added β-NGF), the relative levels of Mos, Emi2, Cyclin E and Cdk2 in these populations were rather similar to those measured in freshly prepared meiotic cells (compare Fig. 4 with Fig. 5B–5C for Mos and Emi2, and data not shown for Cyclin E and CDK2). Culture in the presence of β-NGF enhanced the number of phosphohistone H3 labeled MPII from 1.24±0.12/mm to 1.71±0.13 (p<0.001 n = 7) as expected [4], but also the proportion of cells with high levels of Mos; this latter was much more marked in middle to late PS, PS in division, SII and SII in division than in the young PS and RS populations (Fig. 5A). In five different experiments β-NGF treatment induced a significant increase of mean Mos levels in middle to late PS: 99±20 vs 75±15, (p<0.02), PS in division: 64±40 vs 43±29, (p<0.05) and SII: 48±12 vs 37±10, (p<0.05), but did not modify these levels in young PS and RS populations, and not significantly in SII in division (Fig. 5B). Emi2 levels were also increased in late meiotic cells by β-NGF treatment (fig. 5C). On the contrary, β-NGF modified neither Cyclin E nor Cdk2 levels in cultured meiotic cells (n = 3) (data not shown).

## Discussion

### The four identified constituents of CSF are present in male rat meiotic cells

For Mos, Emi2, Cyclin E and Cdk2, both the proteins and their corresponding mRNAs were highlighted in freshly elutriated PS and RS. More importantly, we could quantify by flow cytometric analysis the relative cellular contents in these proteins at every stage of differentiation of meiotic cells, including SII which cannot be isolated by current methods but cytometry. As the cell contents in these proteins culminated just before the first meiotic division, a role of some of these proteins in meiotic divisions could be expected.

It should be emphasized that the Western blotting and the RT-PCR methods, as used in the present study to detect Mos, Emi2, Cyclin E and Cdk2 proteins and mRNAs, are qualitative, but only semi-quantitative, methods. Hence, no definite conclusion about their relative levels between the different samples can be made using these methods. This also holds true for a comparison between the relative levels observed by FACS analysis (on a per cell basis) and those obtained by Western blotting (on a protein-amount basis). Moreover the elutriated PS fraction, analysed by Western blot is a heterogenous population made of: early PS, middle PS and late PS/Diplotene Spermatocytes (see materials and methods section). On the contrary, flow cytometric analysis allowed an accurate measurement of the relative Mos, Emi2, Cyclin E and Cdk2 contents of the different types of meiotic cells (see materials and methods section).

In the case of Mos, our results are in general agreement with, and complete, previous studies. mos mRNA was first detected by Northen blot in mouse RS [22]–[24]. The fact that these authors found mos mRNA only in post meiotic cells is in line with our results showing a higher amount of mos mRNA in RS than in PS and to the less sensitivity of Northen blot compared to that of RT PCR. Subsequently, Mos mRNA and/or protein were found in rodent and frog PS and rodent RS [14], [25] and in rat late PS in culture [26]. However, in disagreement with our results, van der Hoorn et al.[14] saw the 43kD c-Mos protein only in rat PS cells, but not in RS. No definite explanation can be given for this discrepancy, but it has to be underlined that the RS fractions used in the present study were, at more than 8O%, composed of 1C cells (RS cells) (see materials and methods section). Moreover, the presence of Mos in cell-sorted RS (see fig. 4a) fits our western blot results. We observed that Mos was present throughout the long prophase of meiosis I (MI) and that its level culminated in middle to late PS, then decreased after the first meiotic division. Our results appear therefore in agreement with Nagao [27] who observed that a gradual increase of spermatid formation occured a few days after the beginning of the c-Mos protein expression in vitro and suggested that the 43kD c-Mos protein has a role in spermatogenic cells that are close to meiotic division. However, these changes appear rather different from those observed in Xenopus oocytes, in which Mos is synthesized around the time of completion of MI and is at its maximal level during meiosis II (MII) [28] (see discussion below).

To our knowledge, Emi2 and its mRNA have been described before only in the whole mouse testis, and their quantities were found to be higher in the testis than in the ovary [9]. This difference might be due to the presence of Emi2 and its mRNA in a larger number of meiotic cells in the testis than in the ovary. Indeed, our results showed the presence of Emi2 in all rat testis meiotic cells with the highest level in middle to late PS, whereas in the oocyte, Emi2 is only synthesized around the end of MI, just before interkinesis, and it is present at a high level only during MII [29].

Cyclin E and Cdk2 were co-localized in PS and RS, and these two proteins were already present in young PS with their levels culminating in middle to late PS, just before the first meiotic division. Our data are in agreement with previous studies showing the presence of Cdk2 in mouse spermatogonia and spermatocytes [30], [31] and Cyclin E in rat PS and RS [18]. However, Ravnik and Wolgemuth [31] did not detect the presence of Cdk2 in mouse RS. This might be explained by a species-specific staining pattern or, more likely, by the difference of sensitivity of the method used for the antibody revelation: DAB staining and observation on a microscope under bright-field optics in the work of Ravnik and Wolgemuth, scanning fluorescence using a confocal laser unit in our work. Indeed, it appears from our flow cytometric analysis that the levels of Cdk2 are rather low in RS. Our results showed the presence of Cyclin E and Cdk2 in all meiotic cells; this is different from those described in Xenopus oocytes, in which Cyclin E/Cdk2 were present at very low levels during MI and increased markedly just before MII [7].

Taken together our results indicate that the components of CSF are present at high levels in rat spermatocytes at earlier stages of meiosis than in the oocyte. The presence of these four proteins throughout the long prophase of MI might compensate the short duration of MII (stage XIV lasts only 14 hours) which would not be sufficient for the establishment of a CSF-like arrest. A second explanation could be the different fates of the oocyte and of the SII, both blocked in MPII. Whereas in the oocyte, the establishment of CSF activity at the end of MI and its maintenance during prometaphase II and MPII make the oocyte staying alive and blocked until fertilization (at least 48 hours), the blocked SII will probably die quickly [32], [33], stopping RS formation [4].

A further interesting issue is how the meiotic arrest can be achieved at MPII but not at metaphase of meiosis I (MPI) under these conditions. No clear answer can be given at the present time, but it has to be noted that also in the oocyte, many components of the Mos pathway are present and active at MPI. For example, Mos, MAPK, p90rsk (the 90-kD ribosomal protein S6 kinase) and Bub1 (budding uninhibited by benz-imidazole 1) are essential for suppressing the S phase between the first and the second meiotic divisions, yet do not block eggs at MPI [34].

### Cellular contents in Mos and Emi2 are enhanced by β-NGF in late spermatocytes

Under our culture conditions, β-NGF induced a decrease in the yield of MII by blocking SII in MPII together with an increase of Mos in middle to late PS, PS in division and SII ([4] and present results). It should be emphasized that the relatively high standard errors of the mean levels of Mos observed sometimes, both in basal and treated cultures, were due to variations between the different experiments (this is not unexpected as it was a relative quantification: see materials & methods section). Indeed, in all five experiments performed in the presence of β-NGF, all exhibited increased levels of Mos in middle to late PS, PS in division and SII as compared to their corresponding cells under basal condition, resulting therefore in statistical significant differences.

Even under basal condition (no β-NGF added), some middle to late PS, PS in division and SII (roughly 30–40% of the total populations) exhibited high level of Mos (close to those expressed by most of the cells cultured in the presence of β-NGF; see Fig. 5A). This resulted most likely from the action of “endogenous” β-NGF which is produced by (and acts on) the meiotic cells in co-cultures of PS with Sertoli cells [4]. Similarly, the relatively high levels of Mos observed in freshly prepared middle to late PS (see Fig. 4A) would be due to β-NGF produced by meiotic cells in vivo [4]. Actually, the yield of meiosis in vivo is only two RS from one PS [35]. Conversely, even in the presence of a maximal concentration of β-NGF [4], not all middle to late PS, PS in division and SII exhibited high Mos levels (see Fig. 5A). This fits with our previous results [4] demonstrating that under the same conditions (a maximal concentration of β-NGF) the number of RS formed in vitro is decreased at most two fold as compared to cultures performed under basal conditions.

Overexpression of the proto-oncogene v-mos (pp39^v-mos^) during male meiosis results in a “CSF-like arrest” at MPI [36]. However overexpression of v-Mos occured in leptotene-zygotene spermatocytes, i.e. much before middle to late PS. This could explain, at least in part, that the blockade was observed at MPI and not at MPII.

The physiological regulation of Mos synthesis is not well-understood. However, a positive feed-back between MAPK and Mos synthesis has been described in the oocyte [37]. This is of interest since one pathway of neurotropin action involves the MAPK [38] which are present, together with NGF receptors, in both Sertoli cells and late spermatocytes [4], [12].

Since Emi2 was also up-regulated by β-NGF in late meiotic cells, it is tempting to speculate that these proteins are involved in the same pathway of regulation of spermatocyte differentiation as they are in the oocyte [8], [9], [34]. As there is an increase in Mos and Emi2 in the oocyte at the time of its arrest in MPII [28], [29], our results strongly suggest a relationship between the increase of Mos and Emi2 induced by β-NGF and the β-NGF induced arrest of SII in MPII. Conversely Cyclin E and Cdk2 are not likely involved in the blockage of SII as neither protein was increased under β-NGF treatment. Besides it must be underlined that in the oocyte stable CSF arrest may occur only after full inhibition of the anaphase promoting complex induced by the two pathways [34], which would not be necessary in the blocked SII which will degenerate quickly.

By negatively regulating the meiotic differentiation of PS, NGF could be implicated in the adjustment of the number of RS supported by Sertoli cells. To ensure an optimal number of germ cells per Sertoli cell, germ cell loss (apoptosis) occurs normally during spermatogenesis [33]. Under our in vitro conditions, neither the total (germinal and somatic) number of cells nor their viability was significantly altered by β-NGF treatment [4]. Moreover the proportion of apoptotic germinal cells, determined by caspase-3 assay, was similar under basal and β-NGF-treated conditions (Perrard M-H. and Durand P., unpublished results). No definite explanation to this result can be given at the present time, but it is likely that the low number of degenerating SII was not highlighted due to the rapid phagocytosis of apoptotic cells. This assumption seems all the more likely, as Sertoli cells are highly phagocytic [32] and fits with previous studies [12], [39], [40].

In a previous work we showed that TGFβ1 was also able to regulate negatively the meiotic differentiation of rat PS by blocking SII in metaphase [41]. Synergism and/or redundancy between local regulatory factors is a characteristic of the spermatogenic process [40], [42]. We have found that there is redundancy, but no additivity, between β-NGF and TGFβ1 on the accumulation of MPIIs [43], and that TGFβ1 also up-regulates Mos in late spermatocytes in co-cultures (data not shown). These results indicate that NGF and TGFβ1 could join up on the same pathway in meiotic cells. It is well established that the intracellular effectors of TGFβ1 signalling are the smad proteins [44], but it has also been shown recently that TGFβ1 activates other signalling cascades including the MAPK pathway in different cell types especially in epithelial cells [44], [45].

Taken together the present results suggest that CSF is not restricted to the oocyte. Moreover, they reinforce the view that NGF is one of the intratesticular factors which regulate negatively the second meiotic division by enhancing Mos (and Emi2) in late spermatocytes and thereby adjusting the number of RS that can be supported by the Sertoli cells.

## Materials and Methods

### Animals

These experiments were performed with 20–23- or 90-day- old male Sprague–Dawley rats. The experimental protocol was designed in compliance with recommendations of the European Economic Community (EEC) (86/609/EEC) for the care and use of laboratory animals.

### Reverse transcription-polymerase chain reaction (RT-PCR)

RNA extraction from total germ cells or from elutriated PS and RS fractions from 90-day-old rats [40] and reverse transcription were performed as described [4]. The purity of the PS and RS fractions was assessed by flow cytometry (see below). For the PS fractions 94±3% of the cells were 4C cells, 3±2% were 2C cells and 1±0·5% were 1C cells, n = 5. For the RS fractions (81±2% of cells were 1C cells, 5±1% were 2C cells and 10±2% were 4C cells, n = 3). The PCR reactions were performed according to the instructions of the GoTaq PCR Core System I kit (Promega). Polymerase chain reactions were carried out on 5 µl from 100 µl of RT mix. Primers for cDNA amplification of either mos or emi2 or cyclin E or cdk2 or 18S (Sigma-Aldrich, St Quentin-Fallavier, France) are shown in Table 1. PCR conditions were: denaturation at 94°C for 1 min, annealing for 1 min (mos, emi2 and cdk2) or 1.5 min (cyclin E) or 30 sec. (18S) at the indicated temperatures (Table 1) and elongation at 72°C for 1 min After the last cycle, cell samples were incubated for an additional 10 min at 72°C. Control PCR with untranscribed RNA, or H_2_O were performed in parallel. The amplified products were electrophoresed in parallel with size markers on 2% Metaphor Agarose gels (Tebu, Le Perray-en-Yvelines, France). No product was seen in the negative controls (data not shown).

**Table 1:** Sequences of the primers used to amplify first-strand cDNAs for mos, emi2, cyclin E and cdk2.

mRNA	Accession no.	oligonucleotide sequence	position	Size (bp)	AT* (°C)	Cycles (n)
mos	X52952	Sense: 5′ -GCACCACGACAACATAATCC-3′	1185–1204	312	57.5	30
		Antisense: 5′CAGCCGAAGTCACTTATCTTAC-3′	1475–1496			
emi2	BC082107	Sense: 5′ -AATGGTCAGAACGAGCAGGCACCA-3′	1922–1945	95	66.2	30
		Antisense:TGTTTACTTCTTAGGTGGGTGAGG-3′	1993–2016			
cyclin E	XM_574426	Sense: 5′CTGACCATTGTGTCCTGGCT-3′	788–807	385	59.8	30
		Antisense: 5′TGTCCAGCAAGTCCAAGCTG	1153–1172			
cdk2	NM_199501	Sense: 5′GGAGAACTTCCAAAAGGTGG-3′	153–172	429	56.8	35
		Antisens: 5′GCCAGCTTGATGGACCCCTCTGC-3′	559–581			
18S	M11188.1	Sense: 5′-CGACGACCCATTCGAACGTCT-3′	347–367	312	58	20
		Antisens: 5′-GCTATTGGAGCTGGAATTACCG-3′	637–658			

*AT: annealing temperature.

### Electrophoresis and Western-blotting

Elutriated PS and RS fractions from 90-day-old rats [40] were lysed by sonication in 50 mM Tris-HCl (pH 7.5), 150 mM NaCl, 1% Nonidet P-40, 1 mM Na3VO4, 10 mM NaF, 10 mM betaglycerophosphate, 2 mM EDTA, and 2% v/v of a protease inhibitor Cocktail Tablets (Roche Diagnostics). Protein concentrations were determined with Coomassie Plus Protein Assay Reagent (Thermos Fisher Scientific, Brebieres, France). SDS–PAGE separation was carried out according to the method of Laemmli [46] on 10% polyacrylamide gels. Fifty micrograms of proteins per well were loaded. After electrotransfer, the PVDF membranes (GE healthcare Bio-Sciences) were stained with Ponceau Red S (Sigma-Aldrich) to verify the blotting of proteins on the membrane for each well. Reactions were carried out in PBS-0.05% Tween. The membranes were incubated with the specific antibodies: a rabbit polyclonal antibody raised against Mos (US Biological, Swampscott, MA), or a goat polyclonal antibody against Emi2 (Everest Biotech, Oxfordshire, UK), or a rabbit polyclonal antibody against cyclin E (Delta Biolabs, Gilroy, CA), or a monoclonal antibody against Cdk2 (Santa-Cruz, Le Perray-en-Yvelines, France) at 1/250, 1/300, 1/750 and 1/200 dilutions, respectively. After incubation with appropriate secondary HRP-conjugated antibodies, detection was performed by chemiluminescence (ECL Plus Western Blotting Detection System from GE Healthcare Bio-Sciences).

### Immunocytochemical localization of Mos, cyclin E, Cdk2 and Emi2 in elutriated meiotic cells

#### Immunocytochemical localization of Mos or Emi2

Freshly elutriated PS and RS from 90-day-old rats [40] were fixed with ice cold 70% ethanol, rehydrated and spread on slides. Cells were permeabilized with 0.05% Triton X100 in phosphate buffered saline (PBS) for 30 min, and incubated overnight at 4°C with either a rabbit polyclonal antibody raised against Mos (US Biological), or a goat polyclonal antibody against Emi2 (Everest Biotech) at 1/25 and 1/100 dilutions, respectively. Cells were then washed three times in PBS and incubated for 1 h with either a Fluorolink^TM^ Cy^TM3^ labelled goat anti-rabbit IgG (GE healthcare Bio-Sciences, Orsay, France) or a R-phycoerythrin (R-PE) labelled swine anti-goat IgG (Invitrogen, Cergy Pontoise, France) at 1/300 and 1/100 dilutions respectively. After washing three times in PBS, cells were mounted in Gel/Mount (Invitrogen).

#### Immunocytochemical co-localization of cyclin E and Cdk2

The protocol was the same as described above but the cells were incubated overnight at 4°C with both a rabbit polyclonal antibody against cyclin E (Delta Biolabs) and a monoclonal antibody against Cdk2 (Santa-Cruz) at 1/50 and 1/25 dilutions, respectively. The two antibodies were revealed by a Fluorolink^TM^ Cy^TM3^ labelled goat anti-rabbit IgG (GE Healthcare Bio-Sciences) and a FITC labelled goat anti-mouse IgG (Stemcell Technologies, Grenoble, France) at 1/300 and 1/20 dilutions respectively.

In control reactions, IgG from the same isotype were used. It was previously verified that the Fluorolink^TM^ Cy^TM3^ labelled goat anti-rabbit IgG did not cross react with the FITC labelled goat anti-mouse IgG (data not shown).

#### Image acquisition

Scanning fluorescence images were acquired using a confocal laser unit (Leica TCSSP2) coupled to a microscope equipped with a X63 oil immersion objective.

### Relative quantification of Mos, Emi2, Cyclin E and Cdk2 in meiotic cells by flow cytometry

This was performed according to the methods previously validated in our laboratory [12], [20], [21]. Total germ cells were prepared from 90-day-old rat [40]. Cells were then fixed with ice-cold 70% ethanol, at −20°C for 24 hours before staining. After washing with PBS, fixed cells were resuspended in permeabilizing buffer (0.25% Triton X-100/1% BSA/PBS) for 20 min on ice, then in blocking buffer (5% fetal calf serum/1% BSA/PBS) overnight at +4°C [12].

Germ cells and somatic cells were discriminated by their differential expression of vimentin [12] (vimentin is only expressed by somatic cells). A monoclonal antibody against vimentin (clone V9, DAKO SA, Trappes, France) was conjugated with a red fluorochrome, Alexa 647, by using a Zenon Mouse labeling kit (Invitrogen). A rabbit polyclonal antibody against Mos (USBiological), or a rabbit polyclonal antibody against cyclin E (Delta Biolabs) or a monoclonal antibody against Cdk2 (Santa-Cruz) was conjugated with R-PE by using a Zenon R-PE either rabbit or mouse IgG_1_ labeling kit (Invitrogen).

Cells were incubated with both the labeled anti-vimentin-Alexa 647 antibody at 1/42 dilution and the R-PE labeled antibody raised against either Mos or cyclin E or Cdk2 at 1/26, 1/45, 1/11 dilutions respectively for 30 min at room temperature. Before analysis, Hoechst 33342 (Sigma-Aldrich, St Quentin Fallavier, France) was added to the labelled cell suspension at a final concentration of 0.12 µg/ml for 40 min on ice.

For negative control, IgG from the same isotypes were conjugated with R-PE by using a Zenon R-PE either rabbit or mouse IgG_1_ labeling kit (In vitrogen).

To measure the relative levels of Emi2, germ cells were exposed to a goat polyclonal antibody against Emi2 (Everest Biotech) at a dilution of 1/100, in blocking buffer overnight at 4°C. After washing, cells were incubated with a R-PE- conjugated swine anti-goat IgG at a dilution of 1/100 in blocking buffer, for 1 hour at +4°C. After washing, cells were incubated with the labelled anti-vimentin-Alexa 647 antibody at 1/42 dilution for 30 min at room temperature. It was previously verified that the R-PE- conjugated swine anti-goat IgG did not cross react with the complex vimentin- labelled anti-vimentin-Alexa 647 antibody (data not shown). Before analysis, Hoechst 33342 was added to the labelled cell suspension as described above. Non immune goat IgGs were used as a control.

### Isolation and coculture of PS with Sertoli cells

Co-cultures of Sertoli cells together with elutriated PS were performed as described [40], [47]. Sertoli cells were isolated from about twelve 20–23-day-old Sprague-Dawley rats by enzymatic digestion and plated in bicameral chambers (area 1 cm^2^; polyester membrane, pores 0·4 µm diameter) (Falcon, Becton-Dickinson, Meylan, France) at a density of about 3×10^5^ cells/cm^2^. Cells were then cultured for 3 days in HEPES-buffered F12/DMEM supplemented with insulin (10 µg/ml), transferrin (10 µg/ml), vitamin C (10^−4^ M), vitamin E (10 µg/ml), retinoic acid (3·3×10^−7^ M), retinol (3·3×10^−7^ M), pyruvate (1 mM), testosterone (10^−7^ M) (all products from Sigma, La Verpillière, France) and ovine NIH FSH-20 (1 ng/ml) obtained through NIDDK and Dr A.F. Parlow (lot no. AFP-7028D). On day 3 of culture (referred to as day 0 of co-culture), PS obtained from five adult Sprague-Dawley rats by centrifugal elutriation [48] were seeded (3×10^5^ cells/cm2) on Sertoli cells. 13% of elutriated PS/DS were early PS (stages XIV–IV), 61% were middle PS (stages V–IX), and 26% were late PS/Diplotene Spermatocytes (stages X–XIII) [40]. In some wells, 10 ng/ml of human recombinant β-NGF (Pepro-Tech, London, UK) were added in both the apical and the basal compartments of the chamber. Only basal media (with or without β-NGF) were renewed every two days; β-NGF was added to the apical media at the same interval [4].

### Immunocytochemical reaction against phosphorylated histone H3

On day three of co-culture, cells were fixed directly in the wells with Bouin's fixative for 20 min at room temperature and incubated with an anti- phospho-Histone H3 (Ser10) antibody (a rabbit polyclonal antibody from upstate, Lake Placid, NY) as described [4]. The number of MPI and MPII was determined by microscopic examination of the whole insert membrane; the results were expressed as the number of MPI or MPII per millimeter of well and on the width of the microscopic field (objective 100X) [4].

### Relative quantification of Mos, Emi2, Cyclin E and Cdk2 in cultured meiotic cells by flow cytometry

On day 3 of co-culture, cells were detached from culture wells by trypsinization. Cells were fixed in ice cold 70% ethanol at −20°C for 24 hours before staining. Then cells were rehydrated and permeabilized [12].

The relative levels of Mos, Emi2, cyclin E and Cdk2 were measured in co-cultured meiotic cells as described above for meiotic cells. Additionally, in order to identify PS in division and SII in division, cells were incubated as above with an Alexa Fluor 488 conjugated (Alexa Fluor 488 labeling kit from Invitrogen) anti- phospho-Histone H3 (Ser10) antibody [12] at 1/12 dilution. It was previously verified that the R-PE- conjugated swine anti-goat IgG did not cross react with the complex phospho-Histone H3- Alexa Fluor 488 conjugated anti- phospho-Histone H3 antibody (data not shown).

### Flow cytometric analysis and cell sorting

After immunolabeling, cells were analyzed using a FACSVantage SE cell sorter (BD Biosciences, CA) equipped with an Enterprise II argon ion laser emitting simultaneously a 50 mw line tuned to UV (351 nm) and a second line tuned to 448 nm at 130 mw, to respectively excite Hoechst 33342 and Alexa Fluor 488 or PE or Alexa 647. Emission of PE (λreem  = 585 nm), Alexa 647 (λreem  = 647 nm) and Alexa Fluor 488 (λreem  = 530 nm) fluorescence were acquired after logarithmic amplification through band-pass filters respectively BP 585/40 nm, 675/40 nm and BP 530/30 nm. Emission of Hoechst 33342 fluorescence (λreem  = 424 nm) was acquired after linear amplification through a 424/44 nm filter. Acquisition and analysis were performed using CellQuest ProTM 4.0.2 software. Cell sorting on slides was performed using CloneCytTM Plus 3.1 software. Analyses were performed as previously described in details [12]. Six data parameters were acquired and stored in list mode files: linear forward light scatter (FSC) and linear side angle light scatter (SSC) which roughly represent cell size and cellular granularity, respectively; logarithmic (log) PE (Mos, Emi2, cyclin E or Cdk2), log Alexa Fluor 488 (phospho-Histone H3), log Alexa 647 (vimentin) to detect the immunolabeling, and linear Hoechst to measure the DNA content of the different populations of cells. Contaminating events such as debris and clumped cells were eliminated from the analysis by gated on morphological parameters. In order to allow the detection of a sufficient number of PS in division [which represented 3.7±0.4% of PS (m±s.e.m., n = 5)] and of SII in division (which represented 5.1±2.7% of SII (m±s.e.m., n = 5)) which were both rare cells [49], each acquisition was performed on 1.8±0.3 millions co-cultured cells [co-cultured cells contained 34±7% Sertoli cells and 66±7% germ cells (m±s.e.m., n = 5)].

### Computer analysis

**Figure 6:**
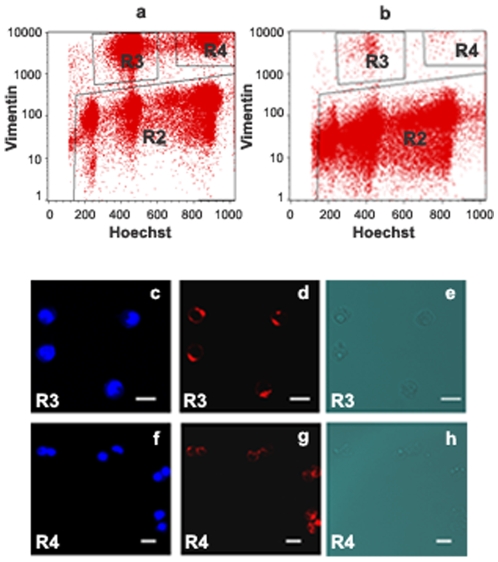
Identification of Sertoli cells and germ cells in co-cultures of PS with Sertoli cells on day 3, by using a monoclonal antibody against vimentin conjugated with a red fluorochrome, Alexa 647. a: Representative flow cytometry analysis of co-cultured cell populations on day 3: Distribution of vimentin-positive diploid Sertoli cells (R3), vimentin-positive tetraploid Sertoli cells (R4), and vimentin-negative germ cells (R2) according to their ploidy (Hoechst 33342). The high specificity of the anti-vimentin antibody was shown by the near complete disappearance of R3 and R4 regions, when co-cultured cells were incubated with non immune mouse IgG conjugated with Alexa 647 as a control (b). Vimentin-positive diploid Sertoli cells (R3) and vimentin-positive tetraploid Sertoli cells (R4) were sorted on glass slides and examined for Hoechst (c, f) and vimentin (d, g) by fluorescent confocal microscopy. e and h are the transmission pictures corresponding respectively to c, d and f, g. Isolated Sertoli cells (c, d, e) and doublets of Sertoli cells (f, g, h) (as highlighted by the double blue spots showing the two nuclei labelled by Hoechst 33342) were identified in R3 and R4 regions respectively. All bars represent 10 µm.

**Figure 7:**
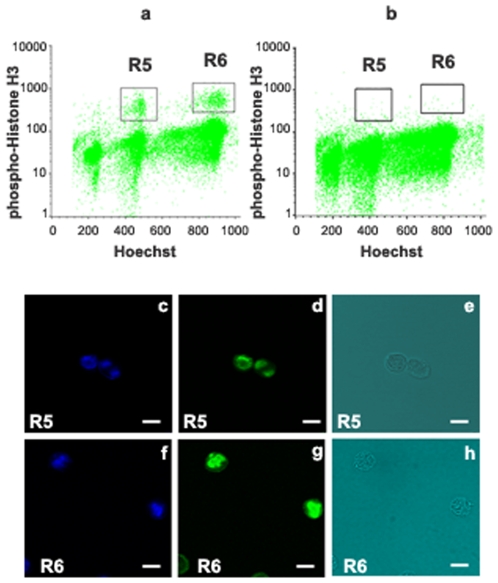
Identification of PS in division (PHH3+ PS) and SII in division (PHH3+ SII) in the germ cell vimentin-negative population (R2 in Fig. 6 ) from co-cultures of PS with Sertoli cells on day 3, by using an Alexa Fluor 488 conjugated rabbit polyclonal antibody against phospho-Histone H3 (Ser10). a: Representative flow cytometry analysis of vimentin-negative germ cell population on day 3: Distribution of PHH3-positive diploid germ cells (R5) and PHH3-positive tetraploid germ cells (R6) (Hoechst 33342). The high specificity of anti- phospho-Histone H3 (Ser10) antibody was shown by the disappearance of R5 and R6 regions, when co-cultured cells were incubated with non immune rabbit IgG conjugated with Alexa Fluor 488 as a control (b). PHH3-positive diploid germ cells (R5) and PHH3-positive tetraploid germ cells (R6) were sorted on glass slides and examined for Hoechst (c, f), phospho-Histone H3 (d, g) by fluorescent confocal microscopy. e and h are the transmission pictures corresponding respectively to c, d and f, g. PHH3+ SII (c, d, e) and PHH3+ PS (f, g, h) were identified in R5 and R6 respectively. All bars represent 10 µm.

Relative levels of Mos, Emi2, Cyclin E and Cdk2 were measured in germ cells which are vimentin-negative (window R2 in Fig. 6a). PS in division (PHH3+ PS) and SII in division (PHH3+ SII) were identified by using the Alexa Fluor 488 conjugated anti- phospho-Histone H3 antibody (windows R6 and R5 respectively in Fig. 7a). Then the three different parameters “FSC/SSC and ploidy” allowed the identification of young PS, middle to late PS, SII and RS[12]. The Mos or Emi2 or cyclin E or Cdk2 content for each of these six populations was obtained by subtracting the fluorescence background from the mean fluorescence of positive cells. The linearity of these assays was checked by plotting the contents of RS, doublets and triplets of RS [21] in Mos (r  =  0.976, p<0.001), Emi2 (r  =  0.997, p<0.001), cyclin E (r  =  0.994, p<0.001) or Cdk2 (r  =  0.977, p<0.001).

### Statistical analysis

Analysis of variance followed by the Tukey Least Significant Difference was used to assess statistical differences between different germ cell populations. Paired Student's t test was used to assess statistical differences between treated cells and their corresponding control cells (Basic–statistics: www.statsoft.com/textbook/stbasic.html and [4].
